# Achieving Permanent Male Infertility by Magnetic Nanoparticle Hyperthermia: A Breakthrough in Animal Fertility Management

**DOI:** 10.3390/pharmaceutics17050602

**Published:** 2025-05-02

**Authors:** Juliana Lis Mendes Brito, Vanessa Nicolau Lima, José Luiz P. R. Jivago, Aline R. M. Marangon, Marcus Vinícius-Araújo, Andris Figueiroa Bakuzis, Juliana dos Anjos Ribeiro dos Santos, Paulo E. N. Souza, Ricardo Bentes Azevedo, Carolina Madeira Lucci

**Affiliations:** 1 Laboratory of Animal Reproduction, Department of Physiological Sciences, Institute of Biological Sciences, Campus Universitário Darcy Ribeiro, Brasilia 70910-900, DF, Brazil; juliana.lis@unb.br (J.L.M.B.); vanessanicolaudelima@gmail.com (V.N.L.); jivago@unb.br (J.L.P.R.J.); imagepet.exames@gmail.com (A.R.M.M.); 2 Institute of Physics and CNanoMed, Federal University of Goiás, Goiania 74884-092, GO, Brazil; mvinicius_1@discente.ufg.br (M.V.-A.); bakuzis@ufg.br (A.F.B.); 3 Laboratory of Electron Paramagnetic Resonance, Institute of Physics, University of Brasilia, Brasilia 70910-900, DF, Brazil; juarsantos.un@gmail.com (J.d.A.R.d.S.); psouza@unb.br (P.E.N.S.); 4 Department of Genetics and Morphology, Institute of Biological Sciences, Campus Universitário Darcy Ribeiro, Brasilia 70910-900, DF, Brazil; razevedo@unb.br

**Keywords:** iron oxide nanoparticles, non-surgical castration, nanocontraception, heating, spermatogenesis disruption, animal population control, male infertility

## Abstract

**Background/Objectives**: Non-surgical neutering strategies have long been pursued for male animals. A previous study from our group showed that magnetic nanoparticle hyperthermia (MNH) applied directly to the testicles is a promising non-surgical sterilization method for male animals, causing testicular atrophy and complete disappearance of seminiferous tubules by the end of a 56 day observation. This long-term study was conducted to verify the long-term efficacy and safety of the method. **Methods**: Wistar rats treated with testicular MNH received an intratesticular injection of a magnetic fluid composed of manganese-ferrite nanoparticles functionalized with citrate (MnFe_2_O_4_-citrate) and were subsequently subjected to an alternating magnetic field. Reproductive parameters and animal health were evaluated by blood tests and abdominal ultrasound for 12 months. **Results**: All MNH-treated animals presented testicular degeneration and atrophy, together with severely reduced or undetectable serum testosterone levels. By the end of the experiment, all but two animals had no identifiable gonads. The only two animals still displaying gonadal-like structures were azoospermic, and histopathology revealed the remaining tissue was non-functional. The procedure was well-tolerated and MNH-treated animals presented no long-term side effects. Hemogram, ALT, AST, urea and creatinine levels were within the normal parameters for Wistar rats over the 12 month period. The liver, spleen, kidneys and lungs had normal structures as revealed by abdominal ultrasound and histopathological exams, with no nanoparticle accumulation in the organs over the long term. **Conclusions**: In conclusion, testicular MNH caused irreversible infertility in rats in a single application, with no adverse effects on general animal health.

## 1. Introduction

Almost all countries worldwide face problems relating to the overpopulation of stray and/or invasive animals. This issue brings ecological and economic challenges, such as the spread of zoonotic diseases, predation on wildlife and waste management [1,2,3]. Population control has therefore become increasingly necessary. Culling cats and dogs is no longer deemed acceptable and has proven ineffective in controlling stray/invasive animal population growth [4]. Due to their high reproductive potential [5], the population recovers in a short time. Animal population control has been proven to be most effectively achieved through neutering/spaying [6,7], in addition to being a more humane alternative. A current approach for the long-term control of both stray and feral animal populations is the so-called Trap-Neuter-Return (TNR) program, which involves creating colonies of sterile animals, thereby slowing their reproductive rate and eventually decreasing animal numbers [8,9,10]. Although the focus of population control has always been on sterilizing females, controlling the fertility of both sexes increases the chances of the program’s success. Each intact male is a potential breeder; thus male sterilization contributes to a decrease in the number of pregnant females [11].

While surgical castration is the technique of choice to neuter male animals, it presents complications, including risk of infection and the need for postoperative care [12,13,14,15], making it difficult to implement on a large scale [16], especially for free-roaming animals. As a result, alternatives to surgical castration have been pursued for decades. Ideally, an effective non-surgical neutering method to control stray/invasive animal populations should be permanent, applied in a single treatment, have no serious side effects and not require any additional care.

The development of an alternative method that is safe, effective and can be applied in a single treatment with the immediate return of the animals to their original location without requiring an observation period, could provide a long-term solution to this problem. Testicular hyperthermia has been studied as a potential method for neutering male animals since the 1970s [17,18,19,20,21], as applying localized heat to the testes can induce infertility by disrupting spermatogenesis. Various methods have been explored, including water-bath, microwave radiation, or ultrasound [17,22,23,24,25,26]. However, these approaches were never successful as the results were only temporary and multiple applications were often required, which is neither desirable nor practical.

Nanocontraception is an emerging concept that involves the use of nanomaterials for contraceptive purposes. Over the past decade, research has increasingly focused on this approach, particularly for males. Several studies have examined the direct effects of nanomaterials [27,28,29,30], while others have investigated the potential of photothermal effects of nanomaterials to induce infertility in male animals [31,32,33]. These methods employing gold nanoparticles, tungsten oxide or copper sulfide nanocrystals have shown promising results. Other possibilities are magnetic heating therapy or magnetic nanoparticle hyperthermia (MNH) which exploit the capacity of specific magnetic nanoparticles to respond to an external alternating magnetic field, thereby producing heat [34]. Although induction heating therapy involving magnetic nanoparticles is not a new strategy, having been mainly applied in cancer therapies [35], its application on gonadal tissue to cause infertility has not yet been fully explored. The MNH-based method used here differs from other hyperthermia approaches to cause infertility in that the heat is generated from the inside-out and is evenly distributed throughout the tissue [36,37]. A study by our research group evaluated the effectiveness of testicular MNH on causing infertility in rats and achieved promising results [38]. This approach allowed for the creation of a localized, homogeneous and controllable heat, sufficient to induce infertility within a short period (56 days) [38]. The present study aimed to investigate the long-term (~12 months) effects of testicular MNH on reproductive and health parameters of treated animals, in addition to nanoparticle biodistribution and toxicity to vital organs.

## 2. Materials and Methods

### 2.1. Animals and Experimental Design

Sixteen 12-week-old male Wistar rats were used, with a mean weight of 240 ± 21 g (205–285 g). The animals were housed in groups of 4–5 in cages enriched with a tread wheel, tunnels and chewing toys. Animals were maintained at 25 °C with a 12/12 h light–dark cycle and ad libitum access to tap water and commercial rat food (Nutrina^®^, São Sebastião, DF, Brazil). All animal procedures were approved by the Ethics Committee on Animal Use, University of Brasilia (protocol number, UnBDOC 71/2019).

The animals were randomly divided into 2 groups: the saline group (*n* = 5), in which all animals received an intratesticular injection of sterile saline solution in both testicles, to act as a control, and the MNH group (*n* = 11), whereby all animals received an intratesticular injection of the magnetic fluid in both testicles and were exposed to the external magnetic field to induce testicular hyperthermia. Animals were evaluated over 345 days.

### 2.2. Magnetic Fluid Characteristics

The magnetic fluid used in this study was the same as the one used in our previous study of the short-term reproductive effects of testicular MNH [25]. Briefly, it is a magnetic fluid composed of manganese-ferrite nanoparticles functionalized with citrate (MnFe_2_O_4_-citrate) synthesized according to [39], with an average diameter of 11.4 nm, dynamic light scattering of 273 nm, PDI (polydispersion index) of 0.21 and a concentration of 54 mg MnFe_2_O_4_/mL.

### 2.3. Experimental Protocol

**Figure 1:**
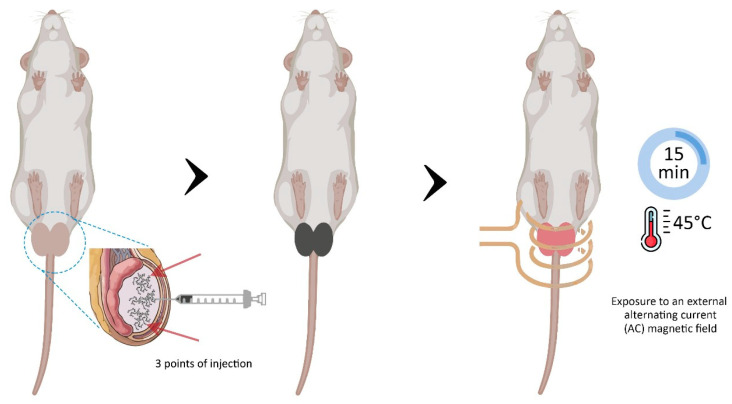
Schematic figure of testicular magnetic nanoparticle hyperthermia (MNH). Animals from the MNH group received a magnetic fluid injection directly into the testicular tissue (150 µL administered equally at 3 different points—syringe and arrows). The testicles were subsequently positioned on a coil and exposed to an alternating magnetic field. Testicular temperature was monitored and maintained for 15 min on reaching 45 °C.

All animals were intraperitoneally anesthetized with ketamine (90 mg/kg) and xylazine (10 mg/kg). After cleaning, each testicle was injected with 150 µL of sterile saline solution (saline group) or the magnetic fluid (MNH group), equally divided into 3 different points of the testicle (top, middle and bottom). The animals in the MNH group subsequently had their testicles positioned on a coil to be exposed to an alternating magnetic field operated at 300 kHz, with an average field amplitude of 240 Oe, according to [40] and as used in [38]. A schematic image of the MNH procedure is shown in Figure 1.

Testicular temperature was monitored using an infrared thermal camera (FLIR SC 620, Wilsonville, OR, USA) and 2 fiber optic temperature sensors positioned on the surface of each testicle. A third fiber optic temperature sensor was inserted into the animal’s rectum to measure rectal temperature. When testicular temperature reached 45 °C, it was maintained for 15 min before the alternating magnetic field was turned off. After the procedure, all animals received a single dose of analgesic and anti-inflammatory (Banamine—1.1 mg/kg SC).

All animals were observed daily for signs of pain [41], behavioral changes and general appearance throughout the experiment. All animals were weighed on the day of the treatment (D0), and then for 10 consecutive days, followed by weekly intervals for the next 3 months and at monthly timepoints until the end of the experiment. Fecal samples were collected daily from each animal over the initial 10 days and frozen for posterior determination of magnetic nanoparticle (MNP) levels. Additionally, blood samples and ultrasound exams (abdominal and testicular) were taken from the animals once a month, spaced on different days to avoid excessive stress on the animals.

After 5 months, 2 animals from the MNH group were euthanized to enable histopathological testicular analysis. The remaining 9 animals in the MNH group and 5 animals from the Saline group were euthanized 345 days post-treatment by anesthetic overdose (ketamine and xylazine), followed by cardiac puncture. The liver, kidneys, spleen and lungs were excised, weighed and divided into 2 parts. One portion was frozen for future MNP level analysis, while the other was fixed for histopathological analysis. Furthermore, when present, the remaining testicles and epididymis were removed, measured (length and width), weighed and processed for histopathological analysis.

The relative weight of the liver, kidney, spleen, lungs, testicles and epididymis was obtained using the formula [(organ weight)/(body weight)*100], and the measurements obtained from the testicles used to determine testicular volume using the mean of the formulas: vol cylinder = [π*(width/2)^2^*length] and vol prolate = [4/3*π*(width/2)²*(length/2)], presented as cm^3^ according to [42].

### 2.4. Ultrasound Examination

Ultrasound examinations were conducted using a veterinary ultrasound device (Z5 Vet, Mindray, Nanshan, Shenzhen, China) equipped with multifrequency probes (5–8 MHz micro-convex and 7.5–10 MHz linear) at monthly intervals throughout the experiment. The evaluations focused on the appearance and size of the testicles and epididymis, together with the appearance of the liver, kidney, spleen, stomach and urinary bladder.

### 2.5. Blood Analysis and Serum Testosterone Test

Animals were anesthetized by ventilation with isoflurane (BioChimico^®^, Rio de Janeiro, RJ, Brazil) in pure oxygen and approximately 1 mL of blood was withdrawn from the jugular vein. A portion of the blood was placed into a Minicollect^®^ tube with EDTA and another portion into a Minicollect^®^ tube with clot activator gel (Minicollect, Araçatuba, SP, Brazil). After blood collection, each animal received 1 mL of saline (S.C.) for fluid replacement.

Whole blood was used for hematological analysis using an automated hematology analyzer (Sysmex pocH—100iV/110iV DiffTM, Kobe, Japan). The parameters evaluated were: red blood cell count (RBC), hemoglobin concentration (HB), hematocrit (HTC), platelet (PLT), white blood cell count (WBC) and lymphocyte percentage (LP) count. After this analysis, the remaining whole blood was frozen for MNP detection.

Serum was used to perform biochemical analyses and the testosterone assay. The biochemical parameters evaluated were alanine aminotransferase (ALT) and aspartate aminotransferase (AST) for liver function, and urea and creatinine for kidney function, using specific assay kits (Labmax 100—Labtest^®^, Santa Lagoa, MG, Brazil) in a biochemical autoanalyzer ChemWell-T (LabTest, Santa Lagoa, MG, Brazil). Total serum testosterone was measured by the enzyme immunoassay technique using Testosterone AccuBind ELISA kits (Monobind Inc., Lake Forest, CA, USA). The hormone detection sensitivity of the test was 0.0576 ng/mL.

### 2.6. Sperm Analysis

Sperm analysis was performed on animals from the saline group and those from the treated group that presented remaining testicles and epididymis. The epididymis cauda was minced in 2 mL of warmed (37 °C) saline solution. A drop of the solution was immediately placed onto a slide for analysis under a light microscope to determine the percentage of motile spermatozoa. The remaining solution was mixed with 10% formalin (1:1) to evaluate sperm morphology and concentration using phase contrast microscopy (Nikon Eclipse Ci, Tokyo, Japan).

### 2.7. Histopathological Evaluation

The testicles and epididymis head were fixed in Bouin’s solution for 24 h, while the other organs (liver, kidneys, spleen and lungs) were fixed in 10% formaldehyde for 24 h. After this period, all organs were dehydrated in ethanol, clarified with xylene and embedded in Paraplast (Sigma Aldrich, St. Louis, MO, USA). Random 5 µm thick sections were obtained from each organ, stained with hematoxylin and eosin and analyzed under a light microscope (Nikon Eclipse Ci-S, Tokyo, Japan). For the testicles and epididymis, the general structure of the organs, organization of seminiferous/epididymal tubules, presence of germline cells and possible changes in the parenchyma and stroma were evaluated. All other organs were evaluated for their general structure and integrity.

### 2.8. Detection and Quantification of Magnetic Nanoparticles (MNP) by Ferromagnetic Resonance (FMR)

To detect and quantify MNP in the liver, kidney, spleen and lungs, the organs were separately minced in distilled water using an Ultra-Turrax^®^ (IKA^®^, Staufen, Germany) until completely homogenized. The homogenized tissues and the feces collected during the first 10 days after the procedure were lyophilized (L101, Liotop, São Carlos, SP, Brazil). The resulting powder was placed into glass capillary tubes, sealed and weighed. The blood samples collected for MNP quantification were directly placed into glass capillary tubes, sealed and weighed. Ferromagnetic resonance (FMR) analyses were conducted using an EMX Plus spectrometer (Bruker Isospin, Bruker, Billerica, MA, USA), equipped with an X-band (9 GHz) high-sensitivity cavity (Bruker ER4119HS, Bruker, Billerica, MA, USA). The FMR analysis used a modulation frequency of 100 kHz, an amplitude modulation of 10 G and 2 mW microwave power, as described in [25].

### 2.9. Statistical Analysis

Prior to the statistical analysis, all data obtained were tested for normality using the Shapiro–Wilk test. Comparisons were made among groups using the ANOVA and Tukey tests with GraphPad Prism 8.0.2 software (GraphPad Software, Inc., San Diego, CA, USA). Differences were considered significant when *p* < 0.05.

## 3. Results

### 3.1. Effect of Testicular MNH on General Animal Condition

**Figure 2:**
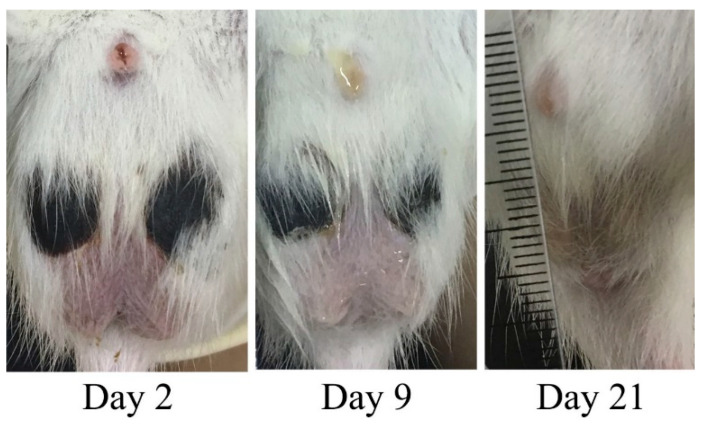
Skin lesions following the testicular MNH procedure in one animal from the MNH group, spontaneously healed after 20 days.

During the 15 min of the MNH procedure, testicular temperature ranged from 44 to 47 °C. Within the first 6 days following the MNH procedure, 81% (9 out 11) of the animals developed skin lesions on the scrotum, which spontaneously healed within 20 days. (Figure 2). The animals did not appear to feel pain or discomfort and showed no behavioral changes.

**Figure 3:**
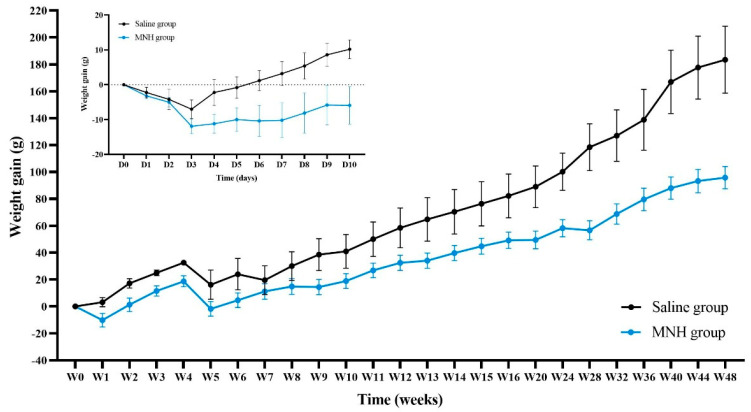
Weight gain (g) of animals in the saline and MNH groups over the first 10 days after treatment (insert) and until the end of the experimental period.

All animals experienced weight loss over the first three days following treatment (7 ± 6 g in the saline group, 12 ± 7 g in the MHT group). Animals in the saline group recovered their initial weight on Day 6 (Figure 3—insert), while animals from the MHT group only recovered their initial weight on Day 13. Subsequently, all animals gained weight as expected (Figure 3).

### 3.2. Reproductive Parameters

#### 3.2.1. Testicular Appearance and Volume

**Figure 4:**
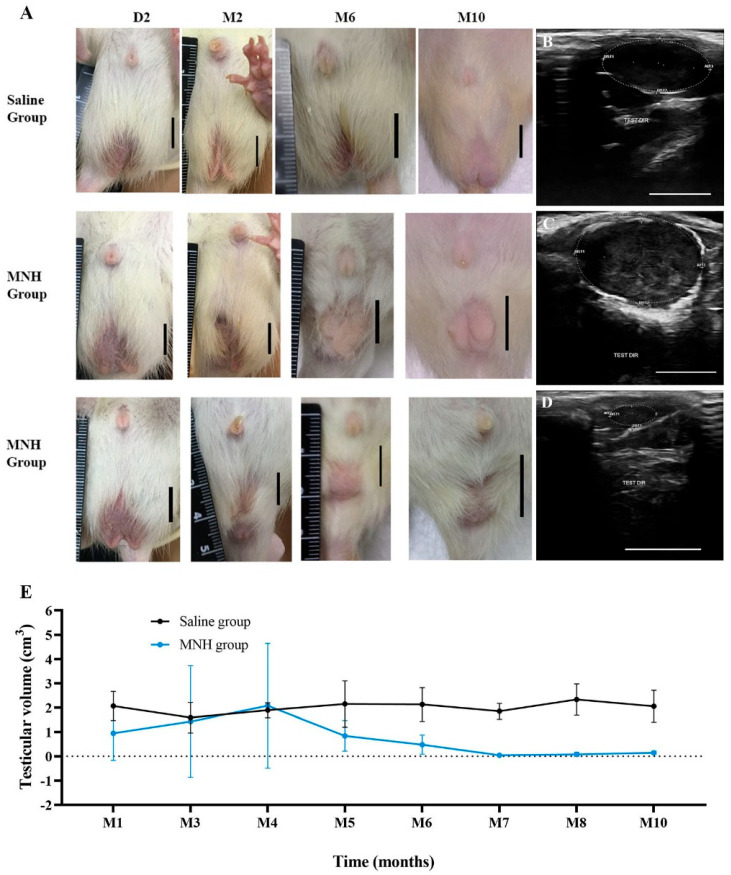
(**A**) Macroscopic appearance of the testicles of an animal from saline group and two animals from the MNH-treated group on Day two (D2), month two (M2), month six (M6) and month ten (M10) after the treatment. While the saline-treated animals did not exhibit any noticeable change in testicular size throughout the experiment period, a number of MNH-treated animals showed enlarged testicles from two to four months followed by a reduction, and others presented a decrease in testicular size as early as the first month post-treatment. A visible atrophy of the gonads was noticed in all MNH-treated animals at M6 which was even more pronounced at M10. Bars = 1 cm. (**B**–**D**) Ultrasonographic appearance of testicles at M4 of the experiment. (**B**) Normal ellipsoid-shaped testicle with regular echogenicity in an animal from the saline group; (**C**) round testicle with regular contours and heterogeneous echotexture in an animal from the MNH group presenting testicle enlargement; (**D**) atrophied testicle with irregular contours and hypoechoic echogenicity in an animal from the MNH group. Bars = 1 cm. (**E**) Mean (±SD) testicular volume (cm^3^) calculated from ultrasonographic examination measurements throughout the experiment for the saline and MNH groups.

The desired castrative effect in MNH-treated animals was first observed as a visible decrease in testicular size (Figure 4A) as early as 25 days after the treatment in some cases (54%—12/22 testicles), while the saline-treated animals did not exhibit any noticeable change in testicular size. Meanwhile, some MNH-treated animals presented enlarged testicles (36%—8/22) from two to four months post-treatment, suggesting local inflammation.

The ultrasonography analysis revealed that all animals from the saline group exhibited normal ellipsoid-shaped testicles with medium echogenicity and homogeneous echotexture and a hyperechoic capsule throughout the study (Figure 4B). In contrast, the MHT-treated animals that experienced testicular enlargement showed rounded testicles with regular contours and coarse echotexture of heterogeneous appearance together with a thick capsule (Figure 4C), while animals with atrophied testicles had very small and hypoechoic structures with undefined contours (Figure 4D).

Testicular volume was measured during the ultrasound examinations (Figure 4E), whereby animals from the saline group showed minimal variation throughout the study period (1.4 to 2.3 cm^3^). Considering all animals, the MNH group exhibited an increase (not significant, *p* > 0.05) in mean testicular volume in the fourth month post-MNH treatment, albeit with considerable variation, as some testicles showed an increase in volume while others showed a decrease. In fact, 11 testicles (50%) completely disappeared within the first four months. After the fourth month, all testicles presented a substantial decrease in volume (maximum volume 0.88 cm^3^), ultimately leading to the complete disappearance of the gonads in almost all MNH-treated animals. By the end of the experiment (345 days), only one MNH-treated animal exhibited the remaining testicles detected by palpation or ultrasound examination, and another presented residual testicular structure only observed at euthanasia.

#### 3.2.2. Serum Testosterone Levels After MNH

**Figure 5:**
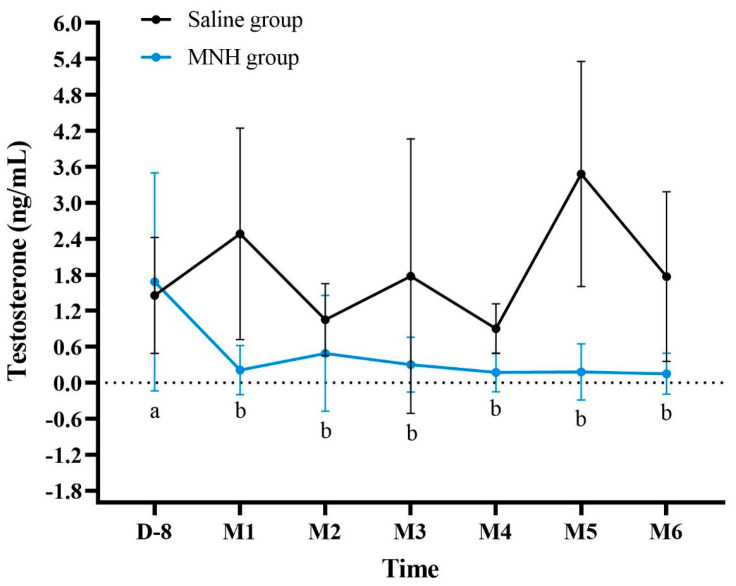
Serum testosterone levels (mean ± SD) in animals from saline and MNH-treated groups. a, b: values with different letters differ (*p* < 0.02). D = day; M = month.

Animals treated with testicular MNH showed a significant decrease in serum testosterone levels as early as 30 days after the procedure (*p* < 0.02) and testosterone levels remained low afterwards (Figure 5). It is noteworthy that 6 months after the procedure, only 2 of the 11 treated animals (18%) presented detectable testosterone levels (0.318 and 1.020 ng/mL). Saline group animals presented mean testosterone levels always >0.9 ng/mL, which were always within the normal limits for the species, with no significant variation throughout the test period (*p* > 0.05).

#### 3.2.3. Sperm Analysis After MNH

At the end of the 345 days, sperm parameters were evaluated in animals that presented testicles and epididymis. All animals from the saline group were evaluated for sperm parameters, with sperm motility of 96 ± 9.7% (mean ± SD), a sperm concentration of 7.21 ± 2.36 × 10^6^ spermatozoa/epididymis cauda and 89.5 ± 5.8% morphologically normal spermatozoa. Only one animal from the MNH group presented a remaining gonadal structure that allowed identification and maceration of the epididymis, and this animal presented azoospermia.

#### 3.2.4. Histological Analysis of Testicles and Epididymis

**Figure 6:**
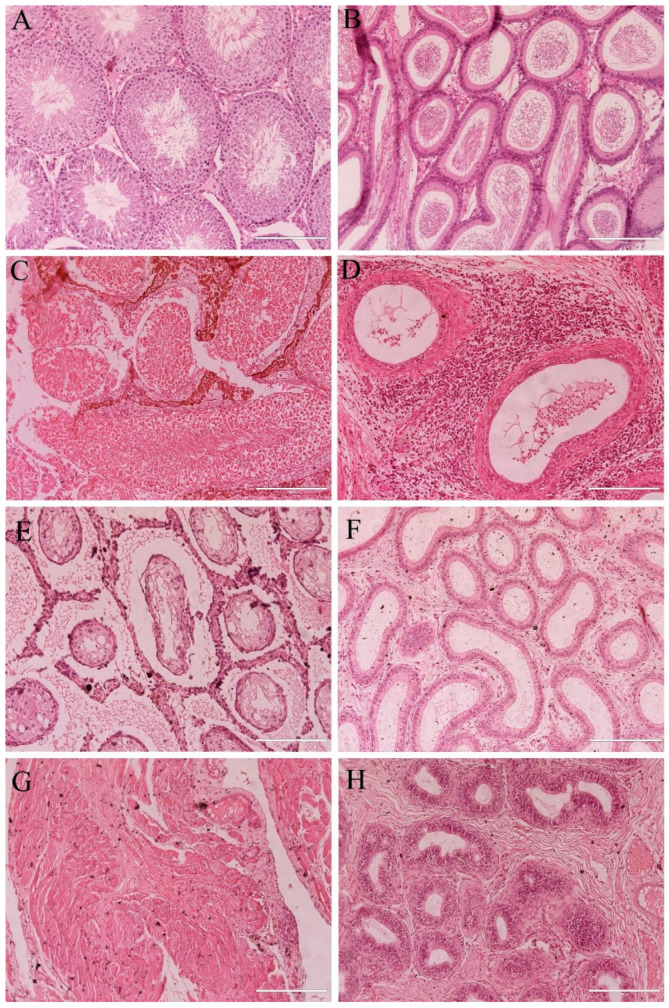
Photomicrograph of testicles and epididymis from the saline group (**A**,**B**); MNH group at month 5 (**C**,**D**), and at month 10 (**E**–**H**). In animals from the saline group, testicles (**A**) presented morphologically normal seminiferous tubule containing germline cells and spermatozoa in the lumen, and epididymis (**B**) showed tubules with columnar epithelium and spermatozoa in the lumen. At month five, MNH-treated animals presented testicles (**C**) with advanced degeneration, coagulative necrosis of seminiferous tubules and epididymis (**D**) showed tubules with flat epithelium and only cellular debris in the lumen and lymphocytic infiltrate on interstitial tissue. On Day 345, the only two MNH-treated animals that still possessed gonadal remnants showed atrophied and highly vacuolated seminiferous tubules and coarsened interstitial tissue on the testicles (**E**) or complete replacement of the testicular parenchyma by connective tissue (**G**), epididymis tubules presented empty lumens (**F**) and cribriform alterations (**H**). Bars = 200 µm.

On day 345 post-treatment, all animals from the saline group presented normal histology of testicles and epididymis. Testicles showed seminiferous tubules close to each other, with seminiferous epithelium with Sertoli cells and germline cells (spermatogonia, spermatocytes and spermatids) in addition to spermatozoids in the lumen (Figure 6A). The interstitial tissue was delicate and contained small groups of Leydig cells. Epididymis tubules presented a simple columnar epithelium with cilia, and the lumen was filled with spermatozoids (Figure 6B).

Of the four testicles obtained from the animals euthanized at M5, one was replaced by a cystic structure (not processed for histology), and another exhibited severely damaged testicular tissue, more specifically with extensive coagulative necrosis (Figure 6C). The epididymis displayed flattened epithelium and debris in the tubular lumen (Figure 6D). In the four remaining gonadal structures from MNH-treated animals at D345, the testicles were highly degenerated, presenting still identifiable seminiferous tubules although atrophied and heavily vacuolated without recognizable germline cells, together with a coarsened stroma (Figure 6E) or showing complete replacement of the testicular parenchyma by connective tissue (Figure 6G). The epididymis tubules exhibited normal epithelium, but empty lumens (Figure 6F) or stratified epithelium, reduced diameter and cribriform alterations (Figure 6H).

### 3.3. Health Parameters

Hematological and biochemical parameters were individually evaluated during the experiment and all animals presented values within the normal limits for Wistar rats, with a few exceptions albeit without important biological meaning. Hematological and biochemical values are presented in Table 1 and Table 2, respectively, for both the saline and MNH groups.

**Figure 7:**
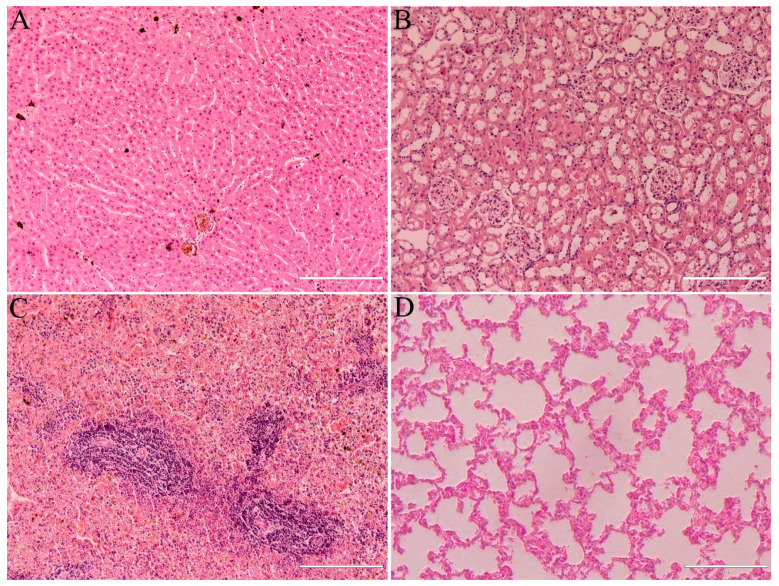
General histological appearance of the liver (**A**), kidney (**B**), spleen (**C**) and lungs (**D**) of MNH-treated animals 345 days post-procedure. Organ structures were similar to the saline group, showing no signs of any pathological process. Bars (**A**–**C**) = 200 µm; (**D**) = 400 µm.

Monthly abdominal ultrasound exams did not reveal any alterations in the organs examined, including the liver, kidneys, spleen, urinary bladder and stomach. The relative weight (%) of the liver, kidney, spleen and lungs did not show any difference (*p* > 0.05) between the saline and MNH groups (Table 3). Furthermore, the histopathological analysis of these organs showed normal structure in all animals from both groups (Figure 7).

### 3.4. MNP Detection by FMR

Nanoparticles were not detected in the organs (liver, kidney, spleen and lungs) or blood of MNH-treated animals after 345 days. The characteristic spectra of magnetic nanoparticles were not observed in the samples, only typical spectra of biological iron radicals such as hemosiderin and ferritin [45,46]. Feces (from the first 10 days post-injection) also did not present signs of nanoparticles.

## 4. Discussion

The present study investigated the long-term efficacy and safety of testicular magnetic nanoparticle hyperthermia (MNH) as an alternative to surgical castration using Wistar rats as an animal model. In our previous study [38], we showed that testicular MNH caused testicular degeneration (as early as 7 days post-treatment) and gradual atrophy for a 56 day period (spermatogenesis duration in Wistar rats—[47]) with no serious side effects. However, for a neutering procedure to be fully acceptable, especially for use in free-roaming animals, it must cause permanent infertility with a single application, not require post-procedure care and not be detrimental to the animal’s health. In the present study, we accompanied animals subjected to testicular MNH for almost one year (345 days). Testicular MNH-treated animals exhibited gradual testicular atrophy accompanied by decreased serum testosterone levels and azoospermia. By the end of the 345 days, only two (out of nine) animals presented remaining testicular tissue which was no longer functional.

Other studies explored the benefits of nanomaterials in promoting infertility in male animals [31,33]. The aforementioned approaches utilized the photothermal effect of intratesticular injections of gold nanoparticles, tungsten oxide or copper sulfide nanocrystals, and showed promising results. These studies showed that the induced heat stress caused a functional disruption of spermatogenesis resulting in male infertility after 14 and 60 days [31,33]. However, all of these studies were performed in mice which have very small testicles. Translating the procedure to the species of interest (i.e., cats and dogs) therefore poses a big challenge, especially when light is to be used, given that light has limited penetration in biological tissues [48]. Rats, on the other hand, have a testicular size similar to cats and small dog breeds. Moreover, even though some adaptations will probably be necessary to apply the method to other species, it is much more probable that the efficacy will be achieved. While near infrared (NIR) light may penetrate 1–3 mm deep in biological tissues [49], a magnetic field has no penetration limit, rendering it advantageous for applications in larger organs/structures. Ding et al. [50] also studied the effects of testicular magnetic hyperthermia in mice, however, employing intravenous magnetic nanoparticle administration. These authors reported critical damage to the reproductive system after 60 days, including partial destruction of the testicles, degeneration of spermatocytes and spermatozoa, when the testicles reached 45 °C.

Besides disrupting spermatogenesis, the induced testicular heat stress can also lead to significantly reduced testosterone levels [51,52,53]. In the present study, MNH-treated animals consistently demonstrated testosterone levels below normal limits as early as the first month post-treatment, and only ~20% of the animals still had detectable testosterone after six months, while the control group had mean testosterone levels within the normal range for Wistar rats [54,55,56]. In contrast, Liu et al. [32] did not observe a significant decrease in testosterone levels in mice subjected to testicular photohyperthermia, suggesting that their treatment preserved secondary sex characteristics in male mice. A significant reduction/absence of testosterone levels could be beneficial, considering its role in animal reproductive behavior. Testosterone significantly impacts behavior, influencing aggression, mood and social interactions [57,58,59,60]. As such, low testosterone levels may lead to a decrease in aggressive behavior, which could be advantageous in managing animal populations.

The histological analysis herein revealed significant damage to the testicular tissue, with a significant portion of the tissue replaced by connective tissue. Our results also corroborate the findings of other studies that showed scrotal hyperthermia can increase germ cell apoptosis, leading to spermatogenesis suppression [32,61]. Notably, in our study, only 2 of the 11 treated animals (18%) retained some testicular tissue after 345 days, and from this, we were only able to analyze sperm in one subject, which presented azoospermia. These findings align with our previous study in rats [38] and reports by [32,33] in mice, in which the testicles shrank in size or completely disappeared following treatment. It is important to emphasize that this neutering approach is strictly intended for animal use only and not for humans in any way.

While the reproductive effects observed in our study were expected and desired, it is crucial to consider additional factors to ensure the overall well-being of the animals. As such, we evaluated the general health of the treated animals over 12 months. MNH-treated animals exhibited a temporary decrease in body weight over the initial three days, which was unconnected to the treatment itself and may in fact be more related to the anesthesia protocol, as reported previously [62], since untreated animals also showed weight loss over the same period. Moreover, the observed weight loss (~10 g in three days) was not biologically important, being considered an acceptable daily variation in rats [63]. Weight gain was maintained afterwards reflecting general health in the long term [64,65,66]. The lower weight gain in treated animals compared to control animals (Figure 2) was probably due to lower testosterone levels.

The only side effect observed in the MNH-treated animals was a skin lesion on the scrotum (in 81% of the animals), occurring two weeks after the procedure and healing spontaneously, with no need for medication. Mild side effects are acceptable and common in any castration method. Surgical castration (orchiectomy) usually causes pain and inflammation of the surgical wound and sometimes hemorrhage and hematoma [67]. Chemical castration methods, such as zinc gluconate intratesticular injection, cause pain, ulceration and an inflammatory process, sometimes worse than the surgical procedure itself [11,68,69]. The procedure described herein caused mild acute side effects, but they were spontaneously solved and did not cause extreme discomfort or pain in the animals. Considering its use in the Trap-Neuter-Return strategy, treated animals could be released immediately after the procedure, without the necessity for further care or medication.

In the long term, all animals remained healthy. Indeed, blood exams evidenced no alterations in the hematological or biochemical (kidney and liver function) analyses, with animals presenting values within the normal limits for the species. Similarly, Soleymani et al. [70] found no significant changes in hematological and biochemical factors following chronic injection of FA@Fe_3_O_4_ NPs at 10, 25 and 50 mg/kg. Moreover, the liver, spleen, kidneys, urinary bladder and stomach did not show ultrasonographic alterations throughout the study period. At the autopsy, 345 days post-treatment, the liver, spleen, kidneys and lungs were macroscopically and histologically normal. Similarly, a study involving mice intravenously injected with four types of iron oxide magnetic nanoparticles with varying diameters showed no histopathological abnormalities in the organs after seven days [71]. Moreover, although the nanoparticles used in this study contain iron, which can accumulate in the liver and potentially lead to cirrhosis and other chronic diseases [72,73], the treated animals showed no clinical signs of iron toxicity at any time during the study. The dose used in the present work (~70 mg NP/Kg B.W.) was sufficient to promote localized heating, but probably too low to cause toxic effects. Overall, our findings prove that the proposed treatment is safe for the animals, with no late or chronic side effects. In fact, the nanoparticles used in this study have previously been shown to have low toxicity when injected into the bloodstream [74] and are even authorized for use in human cancer therapy applications [75].

It is important to consider, however, that the route of nanoparticle administration in the present study (in the testicles) is quite uncommon, making biodistribution crucial to understand, as some may indeed enter the bloodstream. In our previous study showing the short-term (56 days) effects of testicular MNH employing the same nanoparticles, nanoparticles were detected in the liver (5427 a.u.) and the spleen (1014 a.u.) seven days after the intratesticular injection, and subsequently reduced to ~30% and ~20%, respectively, of the initial amount detected 56 days post-injection [38]. Our long-term results proved that no nanoparticles could be detected in the liver, spleen, kidneys, or lungs after 345 days, and that these organs presented normal histological features, thereby proving that the treatment is not toxic as the nanoparticles are eliminated from the body. Another theory that may explain why we did not detect iron oxide in our samples suggests that iron ions can convert into ferritin and hemosiderin iron-protein complexes. These complexes can then transform into transferrin, which is subsequently transported to the bone marrow for hemoglobin production in red blood cells. Alternatively, iron can be utilized to form myoglobin, an iron-protein complex that delivers oxygen to muscles [76].

## 5. Conclusions

In conclusion, this study demonstrates both the efficacy and safety of testicular magnetic nanoparticle hyperthermia as a non-surgical method for inducing permanent infertility in animals. The results show that MNH can cause significant testicular degeneration and decrease testosterone levels, leading to azoospermia and complete gonadal atrophy in most treated animals. The procedure was well-tolerated, with no significant adverse effects observed. Furthermore, the biodistribution of the nanoparticles used did not accumulate in the organs over the long-term. These findings suggest that the testicular MNH method described herein is a promising neutering protocol for population control in animals, warranting translational studies for method application in the species of interest.

## Figures and Tables

**Table 1:** Hematological values (Mean ± SD and variation) in the peripheral blood of animals in the saline and MNH groups at different timepoints.

Saline Group	D8	D15	D30	D60	D90	D120	D150	D180	D210	D240	D270	D300	D330	D345	Normal Limits *
RBC (10^6^/µL)	7 ± 3 (2–9)	9 ± 1 (8–10)	7 ± 1 (6–9)	9 ± 6 (1–18)	9 ± 1 (8–9)	9 ± 0 (9–9)	7 ± 3 (1–10)	10 ± 0 (9–10)	9 ± 0 (8–9)	9 ± 0 (8–9)	9 ± 0 (9–9)	9 ± 0 (9–9)	9 ± 0 (8–9)	8 ± 0 (7–9)	5–10
HB (g/dL)	14 ± 6 (3–18)	17 ± 1 (16–18)	13 ± 2 (10–17)	17 ± 2 (14–18)	17 ± 1 (16–18)	18 ± 0 (18–19)	14 ± 7 (2–18)	19 ± 0 (19–19)	17 ± 0 (17–18)	17 ± 1 (15–18)	17 ± 1 (16–18)	17 ± 0 (17–18)	16 ± 0 (16–17)	15 ± 1 (14–16)	11–19
HTC (%)	37 ± 16 (10–47)	48 ± 4 (45–53)	37 ± 6 (29–47)	39 ± 19 (6–49)	47 ± 3 (43–50)	49 ± 1 (48–50)	38 ± 18 (7–51)	51 ± 1 (51–53)	45 ± 1 (44–47)	45 ± 2 (42–49)	48 ± 0 (47–48)	47 ± 1 (46–48)	45 ± 1 (44–45)	44 ± 3 (40–47)	35–57
PLT (10^3^/µL)	520 ± 285 (133–805)	583 ± 444 (97–966)	168 ± 175 (51–470)	694 ± 376 (37–956)	496 ± 248 (310–920)	932 ± 33 (877–962)	416 ± 397 (59–1022)	915 ± 74 (823–984)	537 ± 323 (171–839)	835 ± 298 (316–1035)	793 ± 180 (627–1008)	965 ± 53 (885–1025)	988 ± 58 (913–1067)	718 ± 193 (425–928)	200–1500
WBC (10^3^/µL)	8 ± 3 (3–10)	10 ± 3 (7–12)	6 ± 2 (3–8)	10 ± 3 (5–13)	8 ± 1 (7–10)	9 ± 2 (8–11)	6 ± 4 (1–11)	7 ± 1 (7–8)	7 ± 1 (6–9)	7 ± 1 (6–8)	6 ± 1 (5–8)	7 ± 1 (6–9)	8 ± 2 (6–11)	5 ± 2 (3–9)	3–17
LP (%)	67 ± 11 (48–78)	68 ± 2 (66–71)	72 ± 4 (66–77)	71 ± 2 (69–73)	69 ± 5 (61–73)	68 ± 7 (57–74)	68 ± 7 (57–74)	70 ± 3 (67–73)	64 ± 4 (60–71)	54 ± 10 (39–62)	63 ± 5 (57–70)	62 ± 6 (56–71)	55 ± 6 (49–63)	55 ± 14 (45–78)	65–85
**MNH Group**	**D8**	**D15**	**D30**	**D60**	**D90**	**D120**	**D150**	**D180**	**D210**	**D240**	**D270**	**D300**	**D330**	**D345**	Normal Limits *
RBC (10^6^/µL)	8 ± 1 (6–9)	7 ± 1 (4–8)	8 ± 1 (6–9)	8 ± 2 (3–9)	8 ± 1 (7–9)	9 ± 1 (7–10)	8 ± 1 (7–9)	9 ± 0 (9–9)	9 ± 0 (8–9)	7 ± 3 (3–9)	8 ± 1 (6–9)	9 ± 0 (8–9)	8 ± 1 (7–9)	8 ± 1 (6–9)	5–10
HB (g/dL)	16 ± 2 (12–18)	13 ± 3 (8–16)	15 ± 2 (11–18)	15 ± 4 (5–18)	16 ± 1 (13–17)	18 ± 2 (14–20)	16 ± 2 (13–18)	17 ± 1 (16–18)	18 ± 1 (16–19)	13 ± 6 (5–18)	16 ± 2 (12–18)	17 ± 1 (16–19)	16 ± 1 (13–18)	15 ± 2 (12–18)	11–19
HTC (%)	43 ± 6 (31–49)	37 ± 7 (21–43)	41 ± 5 (31–48)	41 ± 10 (15–49)	44 ± 4 (36–49)	47 ± 5 (35–52)	46 ± 4 (38–50)	48 ± 2 (44–51)	47 ± 2 (44–51)	35 ± 16 (14–49)	48 ± 11 (33–75)	47 ± 2 (44–52)	44 ± 3 (39–48)	43 ± 5 (34–49)	35–57
PLT (10^3^/µL)	505 ± 256 (133–857)	493 ± 561 (61–1463)	572 ± 352 (56–998)	559 ± 334 (11–1095)	579 ± 348 (69–1117)	822 ± 283 (163–1144)	712 ± 185 (337–870)	734 ± 328 (135–1260)	805 ± 198 (434–1071)	524 ± 403 (91–957)	750 ± 425 (51–1296)	955 ± 196 (536–1233)	982 ± 376 (226–1697)	676 ± 247 (185–948)	200–1500
WBC (10^3^/µL)	6 ± 2 (3–9)	10 ± 6 (5–23)	10 ± 2 (7–13)	13 ± 9 (2–34)	12 ± 6 (7–26)	14 ± 7 (6–26)	11 ± 7 (5–26)	10 ± 4 (4–20)	10 ± 5 (5–22)	7 ± 4 (2–12)	9 ± 5 (4–21)	11 ± 4 (7–19)	11 ± 3 (7–15)	6 ± 2 (2–9)	3–17
LP (%)	73 ± 10 (47–81)	62 ± 12 (45–77)	59 ± 13 (40–78)	65 ± 14 (39–84)	67 ± 11 (43–82)	60 ± 14 (40–80)	67 ± 8 (52–75)	63 ± 11 (43–75)	62 ± 10 (47–73)	64 ± 9 (51–76)	57 ± 11 (38–70)	54 ± 9 (36–65)	49 ± 12 (24–62)	53 ± 7 (41–65)	65–85

RBC: red blood cell count; HB: hemoglobin concentration; HTC: hematocrit; PLT: platelets; WBC: white blood cell count; LP: lymphocyte percentage. * Normal limits for Wistar rats as reported by [43,44]. D = day; M = month.

**Table 2:** Biochemical values (Mean ± SD and variation) for the liver (ALT and AST) and kidney (creatinine and urea) function in the peripheral blood of animals in the saline and MNH groups at different timepoints.

Saline Group	D−8	D15	D30	D60	D90	D120	D150	D180	D210	D240	D270	D300	D330	D345	Normal Limits *
ALT (U/L)	33 ± 16 (21–59)	57 ± 0 (57–57)	27 ± 5 (20–30)	34 ± 4 (28–40)	34 ± 11 (22–50)	33 ± 5 (28–41)	44 ± 6 (35–50)	56 ± 6 (51–66)	61 ± 7 (52–69)	58 ± 7 (49–64)	61 ± 3 (56–64)	69 ± 7 (58–78)	71 ± 11 (61–86)	62 ± 2 (59–65)	17–224
AST (U/L)	99 ± 26 (73–136)	249 ± 0 (249–249)	178 ± 50 (129–243)	93 ± 26 (64–127)	78 ± 6 (71–85)	90 ± 17 (77–120)	106 ± 8 (94–116)	102 ± 25 (82–142)	176 ± 33 (122–208)	130 ± 41 (100–201)	144 ± 43 (106–203)	118 ± 20 (98–148)	156 ± 42 (118–205)	105 ± 98 (0–217)	63–175
CREA (mg/dL)	0.9 ± 0.2 (0.7–1.1)	0.9 ± 0.0 (0.9–0.9)	0.7 ± 0.0 (0.7–0.7)	0.7 ± 0.0 (0.6–0.7)	0.7 ± 0.1 (0.6–0.8)	0.6 ± 0.1 (0.5–0.7)	0.7 ± 0.0 (0.7–0.7)	0.8 ± 0.1 (0.7–1.0)	0.8 ± 0.1 (0.7–0.9)	0.7 ± 0.1 (0.7–0.8)	0.7 ± 0.0 (0.7–0.7)	0.7 ± 0.1 (0.6–0.8)	0.8 ± 0.1 (0.7–1.0)	0.8 ± 0.0 (0.7–0.8)	0.2–0.8
UREA (mg/dL)	51 ± 5 (44–59)	44 ± 0 (44–44)	42 ± 4 (38–46)	40 ± 4 (35–43)	43 ± 1 (42–45)	52 ± 4 (46–56)	48 ± 4 (45–54)	57 ± 3 (54–60)	53 ± 4 (48–58)	47 ± 5 (41–52)	47 ± 2 (45–49)	50 ± 6 (42–55)	44 ± 3 (40–47)	41 ± 3 (37–44)	26–58
**MNH Group**	**D−8**	**D15**	**D30**	**D60**	**D90**	**D120**	**D150**	**D180**	**D210**	**D240**	**D270**	**D300**	**D330**	**D345**	Normal Limits *
ALT (U/L)	32 ± 10 (22–49)	53 ± 24 (28–103)	32 ± 10 (18–50)	47 ± 19 (31–98)	39 ± 8 (25–48)	45 ± 6 (34–53)	68 ± 22 (34–92)	51 ± 12 (38–70)	61 ± 10 (45–76)	71 ± 7 (63–82)	62 ± 4 (57–68)	78 ± 16 (59–113)	78 ± 12 (65–99)	74 ± 6 (67–86)	17–224
AST (U/L)	96 ± 29 (65–156)	190 ± 42 (116–248)	139 ± 32 (102–222)	162 ± 54 (110–298)	128 ± 42 (66–196)	105 ± 32 (58–174)	190 ± 56 (112–291)	153 ± 35 (84–200)	117 ± 17 (93–138)	124 ± 16 (104–149)	138 ± 45 (77–223)	117 ± 28 (89–170)	126 ± 39 (80–219)	125 ± 70 (0–259)	63–175
CREA (mg/dL)	1.0 ± 0.7 (0.6–3.0)	0.8 ± 0.6 (0.0–1.9)	0.8 ± 0.3 (0.6–1.7)	0.8 ± 0.2 (0.5–1.4)	0.7 ± 0.4 (0.1–1.5)	0.9 ± 0.5 (0.6–2.4)	0.9 ± 0.5 (0.7–2.2)	0.9 ± 0.4 (0.7–1.9)	0.9 ± 0.4 (0.7–2.1)	0.9 ± 0.4 (0.7–2.0)	0.9 ± 0.4 (0.7–2.0)	0.8 ± 0.5 (0.5–2.1)	0.8 ± 0.4 (0.6–1.9)	0.9 ± 0.6 (0.6–2.4)	0.2–0.8
UREA (mg/dL)	42 ± 5 (33–49)	50 ± 5 (42–56)	44 ± 6 (31–53)	46 ± 7 (38–64)	44 ± 6 (34–51)	52 ± 12 (39–79)	44 ± 5 (40–55)	47 ± 5 (41–55)	47 ± 7 (34–59)	42 ± 4 (33–47)	39 ± 4 (32–46)	42 ± 5 (33–48)	41 ± 4 (32–47)	39 ± 5 (30–50)	26–58

ALT: alanine aminotransferase; AST: aspartate aminotransferase; CREA: creatinine. * Normal limits for Wistar rats as reported by [43,44]. D = day; M = month.

**Table 3:** Relative weight (%, mean ± SD) of the liver, kidney, spleen and lungs in animals in the saline and MNH groups.

Organ	Saline Group	MNH Group
Liver	2.8 ± 0.1	2.5 ± 0.4
Kidney	0.3 ± 0.0	0.3 ± 0.0
Spleen	0.2 ± 0.1	0.3 ± 0.1
Lungs	0.4 ± 0.1	0.5 ± 0.1

## Data Availability

The original contributions presented in this study are included in the article. Further inquiries can be directed to the corresponding author(s).

## Abbreviations

The following abbreviations are used in this manuscript:

MNH	Magnetic Nanoparticle Hyperthermia
MNP	Magnetic Nanoparticles
FMR	Ferromagnetic Resonance
ALT	Alanine Aminotransferase
AST	Aspartate Aminotransferase
RBC	Red Blood Cell Count
HB	Hemoglobin Concentration
HTC	Hematocrit
PLT	Platelets
WBC	White Blood Cell Count
LP	Lymphocyte Percentage
